# Beneath the Layers: Deciphering the Molecular Pathways, Therapeutic Avenues, and Neurological Connections of Soft Tissue Sarcomas

**DOI:** 10.7759/cureus.44694

**Published:** 2023-09-04

**Authors:** Chukwuyem Ekhator, Han Grezenko, Zaroon Haider, Usama Ali Cheema, Haseeb Haider, Syed Naveed Mohsin, Maryam Affaf, Sophia B Bellegarde, Saniya Amir, Sahil Kumar, Abdullah Shehryar, Sidra Arif, Muhammad Usman Fareed, Abdur Rehman

**Affiliations:** 1 Neuro-oncology, College of Osteopathic Medicine, New York Institute of Technology, Old Westbury, USA; 2 Translational Neuroscience, Barrow Neurological Institute, Phoenix, USA; 3 Internal Medicine, CMH Lahore Medical College and Institute of Dentistry, Lahore, PAK; 4 Emergency, Buch International Hospital, Multan, PAK; 5 Medicine and Surgery, CMH Multan Institute of Medical Sciences, Multan, PAK; 6 General Surgery, Cavan General Hospital, Cavan, IRL; 7 Internal Medicine, Women’s Medical and Dental college, Abbottabad, PAK; 8 Pathology and Laboratory Medicine, American University of Antigua, St. John's, ATG; 9 Accident and Emergency, Liaquat National Hospital and Medical College, Karachi, PAK; 10 Medicine, Liaquat National Hospital, Karachi, PAK; 11 Internal Medicine, Allama Iqbal Medical College, Lahore, PAK; 12 Urology, Jinnah Postgraduate Medical Center, Karachi, PAK; 13 Surgery, Nishtar Medical University, Multan, PAK; 14 Surgery, Mayo Hospital, Lahore, PAK

**Keywords:** sarcoma subtypes, neurological manifestations, epigenetic changes, genetic aberrations, soft tissue sarcomas

## Abstract

Soft tissue sarcomas (STSs) are a heterogeneous group of malignancies that have long posed challenges in terms of diagnosis, treatment, and management. This narrative review provides a comprehensive exploration into the multifaceted realm of STS, spanning from its historical origins to the latest advancements in research and clinical care. We delve into the molecular intricacies of STS, highlighting the genetic and epigenetic aberrations that drive these tumors. The review emphasizes the neurological implications of STS, a relatively underexplored area, shedding light on the interplay between tumor biology and neural processes. The evolving therapeutic landscape is discussed, with a focus on the promise of targeted therapies, immunotherapy, and precision medicine. A significant portion is dedicated to the patient-centric approach, underscoring the importance of holistic care that addresses both the physical and psychological needs of STS patients. Furthermore, we highlight the gaps in current research and clinical practices, offering insights into potential avenues for future exploration. This review serves as a valuable resource for clinicians, researchers, and the broader scientific community, encapsulating the current state of STS knowledge and pointing toward future directions in this dynamic field.

## Introduction and background

Soft tissue sarcomas (STSs) encompass a heterogeneous group of malignant tumors that originate from mesenchymal tissues. The intricate nature of these tumors, coupled with their rarity, has rendered them a focal point of both extensive research and clinical intrigue.

The historical evolution of the understanding and management of STS is a testament to medical progress. Historically, surgical resection constituted the primary therapeutic intervention for STS, albeit often accompanied by substantial morbidity. However, the landscape has transformed with the advent of diagnostic imaging techniques and insights gleaned from molecular biology. These advancements have ushered in a more sophisticated approach to STS management. Recent investigations have notably illuminated the role of radiation therapy in treating STS, particularly in instances where surgical excision yields inadequate margins. This recognition of radiation therapy's value underscores the field's aspiration to optimize treatment outcomes [1]. Additionally, research has delved into the significance of indeterminate pulmonary nodules in the context of high-grade STS. These explorations have cast light on the metastatic potential inherent in these tumors, underscoring the urgency of robust diagnostic and treatment strategies [2].

STS continues to pose a formidable challenge in the contemporary clinical realm, chiefly due to its multifaceted nature and aggressive behavior. Contemporary efforts to decipher the complexities of STS and develop targeted therapeutic strategies are yielding promising avenues for improved patient care. The emergence of liquid biopsy techniques, with a particular emphasis on detecting exosomes circulating within sarcoma patients, marks a pivotal step toward enhancing personalized treatment modalities. This innovative approach facilitates the identification of actionable molecular markers, thereby enabling a tailored therapeutic approach based on each patient's unique molecular profile [3].

Furthermore, the unraveling of the molecular underpinnings of STS has unshackled novel therapeutic possibilities. In particular, the discovery of defects in homologous recombination repair (HRR) within STS patients has kindled optimism regarding the efficacy of targeted therapies. This revelation offers a tantalizing glimpse into the potential to exploit these molecular aberrations for therapeutic gain [4].

## Review

Molecular insights into STSs

Genetic and Epigenetic Dynamics

STSs represent a diverse and intricate domain in oncological studies, primarily shaped by their deep-rooted genetic and epigenetic foundations. These molecular elements, akin to the individual threads of a vast tapestry, intertwine to narrate the unique story of each STS subtype. Delving into these genetic and epigenetic intricacies is not just a scientific exploration but a crucial step toward the development of targeted therapeutic interventions. By understanding and addressing these specific molecular markers, there's potential to revolutionize treatment strategies, leading to more effective interventions and ultimately, enhanced patient outcomes.

Genetic and Epigenetic Aberrations

STSs are a complex group of tumors, and their development and progression are deeply influenced by genetic and epigenetic changes. One of the most striking examples of this intricate relationship is seen in Alveolar Soft Part Sarcoma (ASPS). This particular subtype of STS has been the subject of much attention due to its unique genetic hallmark: an unbalanced translocation that results in the formation of the ASPSCR1-TFE3 fusion gene. This gene fusion is not just a passive marker; it actively drives the disease by promoting the transcriptional upregulation of MET, a gene known to play a pivotal role in various cellular processes, including growth and migration. The implications of this genetic anomaly are profound, highlighting how specific genetic changes can dictate the behavior of a tumor. But the genetic landscape of ASPS does not stop there. The tumor microenvironment, which plays a crucial role in how cancers grow and spread, has been found to express immune checkpoints like PD-L1 and CTLA-4. These molecules are key players in the immune system's ability to recognize and attack cancer cells, and their presence in ASPS suggests potential therapeutic strategies. Targeting these checkpoints has already revolutionized the treatment of several cancers, and there's hope that similar approaches could benefit ASPS patients [5].

Molecular Pathogenesis and Mechanisms

The molecular underpinnings of STSs present a rich tapestry of events that shape their development and progression. Among the various subtypes, Clear Cell Renal Cell Carcinoma (ccRCC) offers a unique perspective into this intricate world. A defining feature of ccRCC is the somatic genomic alterations observed on the short arm of chromosome 3, specifically 3p. These changes frequently target the von Hippel-Lindau (VHL) gene, a pivotal player in cellular processes. The alterations in the VHL gene in ccRCC serve as a testament to the diverse genetic changes that can influence the trajectory of STS, shedding light on the molecular mechanisms that drive tumorigenesis [6]. Adding to this complexity is the tumor microenvironment of Alveolar Soft Part Sarcoma (ASPS). Notably, ASPS tumors have been found to exhibit a reduced presence of tumor-infiltrating lymphocytes, suggesting potential implications for the tumor's interaction with the immune system and its responsiveness to immunotherapeutic strategies.

Genomic Profiling Unveiling Insights

Genomic profiling emerges as a potent lens to decipher the molecular tapestry of STS. In harmony with this concept, a study linked to the EORTC (European Organization for Research and Treatment of Cancer) 90101 “CREATE” trial offers a symphony of insight. This endeavor conducted a comprehensive molecular analysis of ASPS tissue samples, aiming to unravel potential biomarkers correlating with treatment outcomes. Genomic profiling becomes a beacon, shedding light on potential therapeutic targets and prognostic markers, shaping the course of clinical decisions [5].

Clinical spectrum and diagnostic pathways

Diverse Clinical Presentation

STSs stand as a testament to the vast diversity inherent in oncological conditions. These tumors, stemming from mesenchymal tissues, can have origins as varied as muscle, fat, nerves, connective tissue, and even blood vessels. This wide-ranging origin translates into a broad spectrum of clinical presentations, each with its unique set of characteristics and challenges. The clinical manifestation of an STS is not just a reflection of its tissue of origin but is also strongly influenced by other factors. The specific subtype of the tumor, its anatomical location within the body, and its stage of progression all play pivotal roles in determining how the disease presents and progresses. For instance, a sarcoma originating in the muscles might present differently from one that arises in the blood vessels, even if they are at the same stage. Similarly, an early-stage tumor might be asymptomatic, while a more advanced one could lead to noticeable symptoms. This multifaceted nature of STS underscores the importance of a comprehensive and nuanced approach to diagnosis and treatment, ensuring that each patient's unique clinical picture is accurately captured and addressed [7].

Variety in Clinical Manifestations

STSs encompass a rich tapestry of tumors arising from mesenchymal tissues, ranging from muscle and fat to nerves and blood vessels. The clinical tableau that STS presents is marked by a spectrum of manifestations. Often, STS unveils itself as a painless lump or mass that grows progressively over time. The nature of its presentation may shift based on its anatomical location, potentially culminating in functional impairment or discomfort. A sarcoma nestled within the confines of a thigh could disrupt ambulation, while one situated proximal to the lungs might provoke respiratory distress [8]. Notably, it is imperative to recognize that while many lumps may be benign, the emergence of any novel or expanding mass warrants thorough medical scrutiny.

Precision Through Advanced Imaging Techniques

In the realm of STSs, imaging techniques play an instrumental role, acting as the eyes through which clinicians can visualize and understand the tumor's nature and extent. Among the array of imaging modalities, Magnetic Resonance Imaging (MRI) often emerges as the primary choice for many clinicians. Its unparalleled ability to provide detailed images of soft tissues makes it an invaluable tool, allowing for a comprehensive assessment of the tumor's size, exact location, and its relationship to surrounding structures [9]. However, MRI is not the sole player in this orchestration. Computed tomography (CT) scans, with their ability to provide cross-sectional views of the body, are particularly adept at detecting lung metastases, a common occurrence in advanced stages of STS [10]. Adding another layer of depth to this imaging ensemble is the Positron Emission Tomography (PET) scan. Beyond mere anatomical details, PET scans provide insights into the metabolic activity of tissues, helping differentiate between benign growths and malignant tumors. Moreover, they are instrumental in identifying metastatic hotspots, and areas where the cancer might have spread [11]. Together, these imaging techniques compose a harmonious symphony, each adding its unique note, ensuring a comprehensive and accurate portrayal of STS.

Biopsy As the Gateway to Histopathological Insight

A decisive turn in the diagnostic journey of STS culminates in the biopsy. A varied ensemble of biopsy techniques exists, each contributing to the mosaic of diagnostic insight. Among these, the cadence of fine needle aspiration (FNA), core needle biopsy, incisional biopsy, and excisional biopsy echoes [12]. Once the biopsy baton is passed, histopathological scrutiny assumes the spotlight, deciphering the sarcoma's type and grade. The grade, in particular, unveils the tumor's aggressiveness, a crucial facet guiding therapeutic decisions [13]. The overture of advanced assays, including immunohistochemistry and molecular diagnostics, offers a richer melodic depth, further refining the identity of the tumor and unveiling its unique genetic and molecular tapestry [14].

In Harmony With Clinical Literature

As the chapters of this narrative unfold, it's evident that STSs choreograph a diverse clinical symphony. The clinical manifestations are dictated by the tumor's origin, each unveiling its unique set of challenges. The interplay of advanced imaging techniques enlivens the diagnostic landscape, while biopsies and histopathological examinations bring forth the crescendo of certainty. Through this orchestration, medical practitioners strive to harmonize clinical care with the rich melodies of literature, aiming to navigate the intricate pathways of STS with precision and insight.

Navigating therapeutic landscapes

Traditional Approaches and Efficacy

Navigating the complex landscape of STSs has historically relied on a trio of therapeutic interventions: surgery, radiation therapy, and chemotherapy. Each of these modalities has carved its niche in the management of STS, contributing distinctively to patient outcomes. Surgery, for instance, remains the cornerstone for treating localized STS. The surgical approach is not just about removing the tumor; it is an art that seeks to strike a balance between ensuring oncological safety and preserving the patient's functional integrity. The goal is clear: to remove the entire tumor while ensuring that the patient's quality of life remains uncompromised [15]. Complementing the surgical approach is radiation therapy. Often employed either before (neoadjuvant) or after (adjuvant) surgery, radiation therapy aims to enhance local control, especially in scenarios where the tumor is high-grade or when achieving clear surgical margins is challenging [16]. Lastly, there is chemotherapy, a systemic approach that has shown varied efficacy across different STS subtypes. While certain subtypes like rhabdomyosarcoma respond favorably to chemotherapy, their role in many adult STS remains a topic of ongoing research and debate. However, in situations where the disease has spread (metastasized) or in specific high-risk scenarios, chemotherapy continues to be an essential tool in the therapeutic arsenal [17]. Together, these traditional approaches form the bedrock of STS management, each playing its part in the symphony of care.

Harboring Hope in Targeted Therapies

As the curtain rises on the molecular era, targeted therapies emerge as a star on the horizon. These therapeutic overtures set out to enthrall by directing their spotlight to specific molecular abnormalities dancing within the tumor's genetic symphony. The tune of imatinib, a tyrosine kinase inhibitor, takes center stage. It has unveiled its efficacy in gastrointestinal stromal tumors (GISTs) that house c-KIT mutations [18]. However, the complexity of STS unravels challenges-heterogeneity casts shadows over the quest to unveil actionable targets across all subtypes. As the overture of targeted therapies resonates, so too does the prospect of resistance, necessitating the orchestration of combination approaches or the introduction of new players [19].

The Rising Crescendo of Immunotherapy and Precision

Immunotherapy, particularly the echoes of checkpoint inhibitors, has ignited a revolution across the landscape of oncology. In the world of STS, the opening strains of promise have been heard in early trials, particularly among subtypes like undifferentiated pleomorphic sarcoma [20]. In harmony with these developments, the concept of precision medicine ascends. It capitalizes on the individual patient's tumor genetics, shaping treatment strategies accordingly. Molecular profiling steps onto the stage, guiding the selection of targeted therapies or immunotherapies fine-tuned to the unique signature of each patient's tumor [21].

In Harmony With Clinical Literature

In the symphony of STSs, the therapeutic horizons are marked by a dance of traditional modalities and emerging orchestrations. Surgery, radiation, and chemotherapy remain as the stalwarts, while targeted therapies compose new melodies. The harmony of immunotherapy and the cadence of precision medicine paint a promising future, each striving to enhance the notes of patient care. As the maestro of medicine waves the baton, the intricate symphony of STS advances, aiming to harmonize progress with the echoes of the clinical score.

Neurological interplay in STS

Intricate Neurological Tapestry

STSs craft a diverse tapestry, where the threads of neurology intricately intermingle. These tumors, when positioned near or infiltrating neural structures, can unveil a spectrum of neurological manifestations. A symphony of neuropathic pain, numbness, tingling, and even motor deficits can emerge, their composition dependent on the nerve ensnared [22]. Additionally, the overture of metastatic STS, although uncommon, can resonate with a symphony of neurological symptoms-seizures, cognitive shifts, and sensory or motor deficits when the brain or spinal cord becomes an unwitting stage [23].

Molecular Threads Stitching STS to Neural Origins

Recent research endeavors have begun to unveil the intricate molecular links that tether STS to neural processes. Within this evolving narrative, synovial sarcoma emerges as a notable figure, elegantly expressing neural markers that whisper of a potential origin or differentiation rooted in the neural crest [24]. Moreover, genetic mutations and pathways that unravel within STS might also be instrumental players in neural development or function. A spotlight shines on the EWSR1-FLI1 fusion gene, characteristically adorning Ewing's sarcoma. Its resonance reverberates through both tumorigenesis and neural differentiation [25].

Nurturing Implications for Treatment and Care

The neurological interplay within STS sketches far-reaching implications across the landscape of treatment and patient care. In scenarios where STS ventures to invade neural domains, the surgical tableau becomes intricate, striking a harmonious balance between tumor eradication and safeguarding neurological function [26]. Furthermore, the potential neural metamorphosis of select STS subtypes may unfold avenues for targeted therapies, where molecular pathways could be harnessed to craft resonant interventions. Within this symphony, the understanding of neurological nuances nourishes the choreography of symptom management, rehabilitation, and ultimately, the melodic crescendo of enhanced patient quality of life [27].

Harmony in Progress

STSs present a unique interplay with the realm of neurology, adding layers of complexity and depth to their narrative. The relationship between these tumors and the nervous system is multifaceted, influencing not only the clinical presentation but also the diagnostic and therapeutic strategies. As STS tumors grow and evolve, they may interact with nearby neural structures, leading to a range of neurological symptoms and challenges. This intricate dance between the tumor and the nervous system necessitates a nuanced approach, ensuring that both oncological and neurological aspects are addressed in tandem. The evolving insights into the molecular underpinnings of STS further enrich this narrative, promising more targeted and effective interventions in the future. As the field of oncology continues to advance, there is a palpable sense of optimism. The hope is for a future where the complexities of STS are not just understood but also effectively managed, ensuring that patient care is not only precise but also imbued with empathy and understanding.

Multidisciplinary approach to STS

Strategic Ensemble for Complex Crescendos

The journey through the intricate landscape of STSs demands not just a soloist, but a harmonious ensemble of specialists. A multidisciplinary approach emerges as the symphony that orchestrates optimal diagnosis, treatment, and follow-up. In this symphony, various virtuosos wield their expertise to compose a holistic strategy. By weaving the insights of different specialists-each with their instrument of knowledge-a comprehensive composition resonates, considering not only the tumor's anatomy and characteristics but also the patient's holistic context and preferences [10]. This collaborative symphony aligns all aspects of care, from inception to recovery, transmuting into improved survival rates and enhanced quality of life.

Expertise En Pointe

In the composition of STS care, various specialists perform essential roles. Surgeons emerge as maestros, particularly during the intricate act of tumor resection. Their virtuosity shapes the depth of surgical excision, deftly balancing complete tumor removal with the preservation of function and aesthetics. Meanwhile, the spotlight falls on medical oncologists, conductors of systemic treatment. Under their baton, chemotherapy, targeted therapies, and immunotherapy harmonize. They diligently assess the need for pre- or post-operative interventions, orchestrating vigilant monitoring of treatment effects and patient response [28]. Radiologists, the interpreters of the body's imagery, lend their expertise in diagnosis, staging, and follow-up. With MRI, CT, and PET scans, cast light on the tumor's dimensions, exact location, and any shadowy traces of metastasis [29]. And when certain STS types flirt with neurology-nestling near or entwining neural structures-neurologists waltz onto the stage. They deftly assess, manage, and navigate the neurological manifestations, ensuring harmony between the tumor's progress and the patient's neural function [30].

In the grand score of STS, the notes of a multidisciplinary symphony resonate, ensuring that patient care is not just a solo performance, but a harmonious ensemble. The delicate balance struck by surgeons, the resonant interventions of medical oncologists, the interpretive prowess of radiologists, and the neurological nuances addressed by neurologists - these themes collectively craft the orchestration of excellence in STS care.

Patient-centered care and quality of life

The Role of Rehabilitation and Supportive Care

In the intricate world of STSs, the focus of care is not solely on the tumor. Instead, it is a holistic approach that views the patient in their entirety, recognizing the myriad facets of their well-being. This philosophy of patient-centered care paints a broad picture, one that goes beyond the confines of medical interventions and delves into the realms of rehabilitation and supportive care, ensuring a comprehensive healing journey.

Following surgical procedures or other treatments, the emphasis shifts to rehabilitation. This phase is crucial, acting as a bridge that helps patients transition back to their daily lives. It is a carefully curated blend of physiotherapy, occupational therapy, and other specialized programs, all tailored to meet the unique needs of each patient. The goal is not just to restore physical strength and mobility but to rejuvenate the very essence of daily living, allowing patients to embrace life in its fullness once again [31].

But the journey of care does not end with rehabilitation. Supportive care steps in, acting as a constant companion that ensures patients' comfort and well-being throughout their healing process. This includes a spectrum of services, from managing pain and addressing potential complications like lymphedema to offering guidance on nutrition. Each element of supportive care plays a symphonic note, enhancing the overall quality of life for patients, ensuring they not only recover but thrive, even in the aftermath of their treatments.

Psychological Implications and Support for STS Patients

The odyssey of an STS patient traverses realms not confined to the corporeal. It is a symphony woven with threads of emotions, anxieties, and reverberations of the psyche. The diagnosis of STS casts a profound shadow, summoning anxiety, depression, and the resonance of fear. This emotional undercurrent is a facet that demands equal consideration. It is here that recognition of psychological implications takes center stage, and the provision of support unfurls as a vital overture. Counseling emerges as a sanctuary where patients' feelings echo, support groups offer shared notes of resilience, and psychotherapy becomes a journey toward emotional equilibrium. Each strand of support provides a symphonic cadence, a counterpoint to the challenges, and a score for the journey's emotional landscape [32].

In the mosaic of STS care, rehabilitation, and supportive care emerge as the symphonies that amplify the resonance of holistic well-being. Beyond the medical choreography, these elements are the heartbeats of quality of life. As the composition unfolds, physical restoration and psychological nourishment harmonize-a crescendo that orchestrates the holistic well-being of the STS patient.

Future directions

Current Gaps in Research and Clinical Care

As we stand on the cusp of progress in the realm of STSs, there are constellations of challenges that await both researchers and clinicians to chart new courses. The first enigma lies in the very tapestry of STS with over 50 histological subtypes, each with its genetic signature and choreography. This complexity, a testament to nature's artistic diversity, presents a formidable challenge in weaving standardized treatment paradigms [33]. Therein lies the beacon for future research and clinical innovation-to harmonize this diversity into a symphony of tailored therapies. Even as targeted therapies illuminate the horizon, some subtypes languish in the shadows of untargeted chemotherapy, beckoning for tailored interventions with diminished side effects [34]. Clinically, the compass must lead to multidisciplinary care - a symposium of medical, surgical, and supportive disciplines, crafting a comprehensive narrative for patient well-being.

Potential Avenues for Future Research

In the crucible of future STS research, the crucible glows with the promise of breakthroughs. The genome's tapestry is being unraveled, revealing tapestries of molecular targets, and laying the groundwork for novel therapies [14]. And as the veil lifts on the tumor microenvironment, a new horizon unfolds, embracing the potential of immunotherapeutic vistas. The script of resistance, a villain in the narrative, is under scrutiny-revealing pathways to surmount its barriers or prevent its inception. The pantheon of diagnostics, equipped with advanced imaging and tools, raises a clarion call for early and precise diagnosis, a cornerstone that can redefine treatment landscapes [35].

Concluding Thoughts on the Evolving Landscape of STS

The journey through the world of STSs is akin to navigating a dynamic and ever-changing terrain. As we tread this path, the guiding principle remains unwavering: the well-being and needs of the patient are at the heart of every decision and intervention. This patient-centric ethos is bolstered by interdisciplinary collaborations, where experts from diverse fields come together to offer a holistic approach to care. The commitment to research is evident, with continuous investments aimed at unraveling the mysteries of STS and devising innovative treatments. Personalized care, tailored to the unique needs and circumstances of each patient, stands as a testament to the dedication and commitment of the medical community [36]. The narrative of STS has evolved over the years, transforming from a singular, one-size-fits-all approach to a multifaceted and nuanced strategy. While challenges persist, they are not insurmountable obstacles but rather stepping stones toward a brighter future. This journey, marked by collective efforts, cutting-edge research, and a deep-seated commitment to patients, paints a hopeful picture for the future of STS care.

## Conclusions

STSs represent a complex array of tumors, each with its unique molecular and clinical signature. This narrative review delves deep into the multifaceted nature of STS, highlighting the challenges and advancements in research and patient care. The vastness of over 50 histological subtypes underscores the intricacy of STS and the necessity for a comprehensive overview. This work serves as a bridge, connecting historical knowledge with contemporary findings, making it invaluable for both seasoned professionals and those new to the field. The emphasis on interdisciplinary collaboration is evident, advocating for a holistic approach that encompasses the entirety of a patient's well-being. Beyond its academic contributions, this review also identifies research gaps and potential avenues for innovation, urging the scientific community to come together in their quest for deeper understanding and improved treatments. In essence, this narrative is more than just a reflection of STS's current landscape; it is a beacon, guiding the way toward a brighter, more informed future in oncology.
